# My body, my choice: Why overruling Roe *vs* Wade is not a pro-life movement

**DOI:** 10.1016/j.lana.2022.100305

**Published:** 2022-06-15

**Authors:** The Lancet Regional Health– Americas

Every year, about 73 million induced abortions take place worldwide, according to WHO, with more than 25 million considered unsafe. It is a simple procedure that can be done surgically or with medication. Provided with medical support, accurate information, and access to medication, women can do it safely in their own homes in the first 12 weeks of pregnancy. In the Americas and Caribbean region, abortion is legal in few countries; one of them is the USA. Since Roe *vs* Wade passed, in 1973, women are theoretically allowed to terminate their pregnancy if they choose so, until 23 weeks, in all 50 US states. But now, after 49 years, a leaked draft opinion written by Justice Samuel Alito rejects and threatens the right to abortion.

Through Roe, the US Supreme Court recognised that abortion is a constitutional right under the 14th Amendment, but states could prohibit abortion after foetal viability. Although it seems that the right to terminate a pregnancy is accessible throughout the USA, overruling Roe has been under motion for some time. For example, from January to November, 2019, 18 states enacted 46 laws that can either restrict or prohibit abortion. Most laws regulating abortion are in the Midwest and South regions. States that have enacted unconstitutional bans are Alabama, Georgia, Kentucky, Louisiana, Mississippi, Ohio, Missouri, Arkansas, and Utah. In 2019, 40 million women lived in hostile states, contrasting with 24 million that lived in supportive states. These numbers have probably worsened in 2021, when a statute to ban abortion after a 6-week pregnancy was enacted in the state of Texas.

Women already suffer consequences without the end of Roe. In 2017, 18% of pregnancies **in the US** ended in abortion, with a total of 862 320 procedures in the USA. That same year, 12% of women needed to travel across state lines from locations considered to have hostile abortion policies. In these states, there are regions called abortion deserts, and patients sometimes need to travel more than 4 h to get to an abortion facility. If Roe is overruled, states will have the power to decide what they want to do in terms of abortion: outlaw it, regulate it, or allow it. Though unconstitutional, the end of Roe could even jeopardise the freedom to travel, as some states plan to ban women from traveling to reach abortion facilities. Some of these hostile states are concentrated in so-called hostile zones, meaning that women will need to cross more than one state to reach a supportive location, thus accessing clinics will be even more challenging for most.

That 75% of women seeking an abortion are of low income and residing in hostile states, makes it even more problematic. A possible solution to the need to travel is abortion pills, which are safe and feasible for home use for those in the beginning of pregnancy. However, the use of these pills is also under threat, meaning that women that live in those states won´t have access to safe and probably timely abortion care. Without proper care and accessibility, unsafe abortion measures will increase, leading to physical health consequences and severe mental health problems. More women may die due to unsafe abortion, and inequalities will be even more pronounced in these regions. 53% of women who needed an abortion in 2014 were Black and Hispanic; in Texas alone, 14% of the population is Black. The poorest in these regions are Blacks (19.5%), followed by Hispanics (17%), and those will be most affected.

One of Justice Samuel Alito's main points for Roe being overruled is the fact that the Constitution does not confer the right to abortion. Yet, Roe is not just a matter of women choosing to terminate a pregnancy. It is a matter of public health, of social class, it is a structural issue related to poverty and race. The US Constitution confers to women the right to privacy, and denying it is a human rights violation that threatens the health and safety of women and girls. When abortion is a choice for women, it saves lives. Banning abortion means taking away the right of women and girls to have proper care, and those that support the ban do not consider that lives are at stake. If this is truly about saving lives, then Roe must definitely not be overruled!

